# Myocardia ischemia associated with a myocardial bridge with no significant atherosclerotic stenosis

**DOI:** 10.1186/s12872-015-0158-2

**Published:** 2015-12-08

**Authors:** Min Yu, Lihong Zhou, Tingting Chen, Kaihong Yi, Chujuan Zeng, Xuerui Tan

**Affiliations:** Department of Cardiology, the First Affiliated Hospital, Shantou University Medical College, Shantou, Guangdong 515041 China

**Keywords:** Myocardial bridge, Electrocardiogram, Coronary angiography

## Abstract

**Background:**

Myocardial bridge refers to the myocardial tissue with which the coronary artery is partly covered. Though it has long been regarded to be benign, patients with myocardial bridges may present with myocardial ischemia, acute coronary syndromes, coronary spasm, sudden cardiac arrest or even sudden death.

**Case presentation:**

In present study, we reviewed four cases with myocardial bridge and no stenosis of coronary artery, which included acute coronary syndrome and sudden cardiac arrest.

**Conclusions:**

These cases indicated that cardiac events in patients with myocardial bridge may be associated with coronary spasm, myocardial supply/demand mismatch or cardiac arrest.

**Electronic supplementary material:**

The online version of this article (doi:10.1186/s12872-015-0158-2) contains supplementary material, which is available to authorized users.

## Background

Myocardial bridge (MB), a common anatomical variant, refers to the myocardial tissue with which the coronary artery is partly covered. It is almost exclusively located in the left anterior descending coronary artery (LAD), rarely in the left circumflex artery and right coronary artery. The frequency of MB varies with different countries and studies [1]. At autopsy the prevalence of MB is reported to be as high as >50 % [1] while the angiographic MB is less commonly observed. Though MB has been regarded as to be benign clinically for a long time, several cardiac events including cardiac arrest, even sudden death caused by MB have been reported. In present study, we reviewed a series of patients with MB presenting with evidence of ischemia and no stenosis of coronary artery. Four case reports were showed. Written informed consent was obtained from the patient for publication of the case reports and any accompanying images.

## Case presentation

### Case 1

**Fig. 1:**
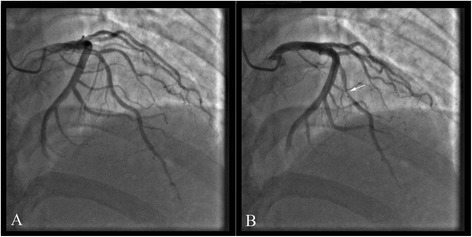
In case 1, coronary angiography with myocardial bridging (MB) of the left anterior descending coronary artery (LAD) during diastole (**a**) and systole (**b**)

A 58-year-old Chinese man came to our hospital because of recurred lasting chest pain at night for 3 days, which was continued about 1 h. The electrocardiogram (ECG) during episodes of chest pain showed ST segment elevation in leads V1–V5 (ECG not obtained). He had a history of smoking for 30 years. On admission, physical examination was unremarkable, except the blood pressure was 152/84 mmHg. ECG showed ST segment depression about 0.03 mV–0.05 mV in leads V5–V6 (ECG not obtained). The myocardial enzyme was normal, the level of troponin I (cTnI) was 0.1 ng/ml (reference, < 0.5 ng/ml), creatine kinase-MB (CK-MB) was 1.8 ng/ml (reference, < 5 ng/ml) and myohemoglobin was 18 ng/ml (reference, < 80 ng/ml). Echocardiography and chest x-ray were normal. Coronary angiography (CAG) demonstrated there was a MB in the middle segment of the LAD (Fig. 1). No stenosis of coronary artery was found.

### Case 2

**Fig. 2:**
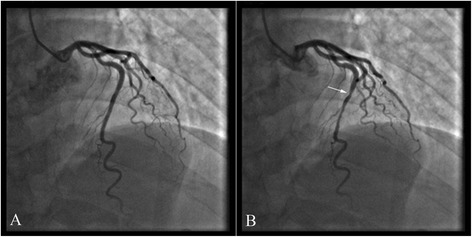
In case 2, coronary angiography showed that a MB in the middle segment of the LAD (**a** diastole; **b** systole)

A 51-year-old Chinese man with a six-month history of angina was admitted to our hospital because of a sudden chest pain lasting about 80 min which reflected to left upper limb and finger side accompanied by diaphoresis. ECG showed ST segment elevation in leads III and aVF (Figure S1 showed in the Additional file 1). Cardiac biomarkers were elevated. The levels of cTnI, CK-MB and myohemoglobin were 6.0 ng/ml, 43.5 ng/ml and 148 ng/ml respectively. CAG showed that a MB in the middle segment of the LAD (Fig. 2). The patient suffered several episodes of chest pain in hospital. ECG showed ST segment elevation in inferior leads (Figure S2 showed in the Additional file 1, ST segment elevation: lead III > lead II). After treatment with nitrates and diltiazem, the chest pain was relieved.

### Case 3

**Fig. 3:**
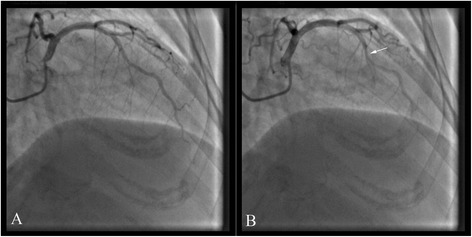
In case 3, coronary angiography showed that a MB in the middle segment of the LAD (**a** diastole; **b** systole)

A 55-year-old Chinese woman was presented to our hospital because of sudden chest pain and shortness of breath for 3 h. She had a one-year history of hypertension. The levels of cTnI, CK-MB and myohemoglobin on admission were 0.5 ng/ml, 11.9 ng/ml and 217.3 ng/ml respectively. The ECG revealed faster atrial fibrillation with a ventricular rate of 110 beats/min and ST segment depression in leads V6 (Figure S3 showed in the Additional file 1). Reexamination of cardiac biomarkers showed that cTnI, CK-MB and myohemoglobin were 5.8 ng/ml, 15 ng/ml and 25.6 ng/ml. CAG showed that a MB in the middle segment of the LAD (Fig. 3).

### Case 4

**Fig. 4:**
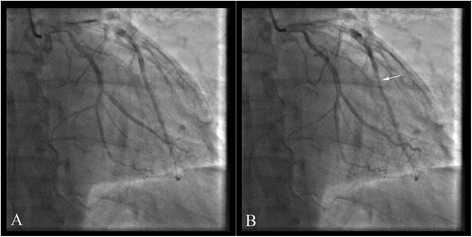
In case 4, a MB in the middle segment of the LAD was showed in coronary angiography (**a** diastole; **b** systole)

A 55-year-old Chinese man was send to our hospital for lasting chest pain for 6 h and sudden cardiac arrest. His past medical history were remarkable for hypertension and diabetes for 3 years. He had a 30-years history of smoking. On admission, his blood pressure was 108/52 mmHg, respiratory rate was 30 breaths/min and his heart rate was 88 beats/min. The pupil diameter was 7 mm on both sides and the light pupillary reflex was disappeared. ECG showed that ST segment depression about 0.01 mV–0.02 mV in leads V4–V6 (Figure S4 showed in the Additional file 1). The levels of cTnI, CK-MB and myohemoglobin on admission were 0.1 ng/ml, 1.8 ng/ml and 125 ng/ml respectively. He suffered recurred cardiac arrest in hospital. CAG showed that a MB in the middle segment of the LAD (Fig. 4). He is doing well at 4 months of follow-up after cardiac arrest.

## Discussion

Since described by Cranicianu in 1922 [2], MB has been widely investigated. Though it has long been regarded to be benign, Ishikawa et al. recently found that MB may be an anatomic risk factor for coronary atherosclerosis and myocardial infarction [3]. Furthermore, MB can lead to myocardial ischemia, infarction, ventricular tachycardia, ventricular fibrillation and sudden cardiac death. In present study, we reviewed the cases with MB and no stenosis of coronary artery, to investigate the underlying mechanisms how cardiac events were caused in the patients who have MB and no stenosis of coronary artery.

Coronary artery spasm, first described in 1959 by Prinzmetal et al. [4], is not an uncommon and has been recognised as an important cause of chest pain in patients with normal or significant obstructive coronary artery. The incidence of spontaneous coronary spasm varied in different countries [5]. It was reported that the incidence of spontaneous coronary spasm was between 0.26 % and 0.93 % during CAG, which may be underestimated owing to nitroglycerin administration, the true incidence may be higher than that is angiographically demonstrable [6]. Compared with those have not MB, coronary spasm at myocardial bridge site was more easily to be provoked by either acetylcholine administration or physical exertion [7]. In case 1, ST segment elevation in V1-V5 leads during episodes of chest pain suggested acute ischemia of LAD. CAG demonstrated no stenosis of coronary artery and MB in LAD, which suggested that spasm in LAD may lead to acute ischemia in patient. In addition, coronary spasm that has not MB can also lead to acute ischemia in patient. Like in case 2, when the patient complained of chest pain, the ECG showed ST segment elevation in inferior leads, which revealed acute ischemia of right coronary. No stenosis of right coronary artery was found in CAG. The ECG changes showed that episodes of chest pain may be caused by spasm of right coronary artery. These cases suggested that coronary spasm may play an important role in acute ischemia in patients with MB.

Acute ischemia occurs when the supply of myocardial blood flow is inadequate compared with the demand. It usually occurs in the setting of coronary arteriosclerosis and coronary spasm. Though MB has long been regarded as a variant without hemodynamic or physiological relevance, recent researches have shown that MB can impair coronary blood flow. Furthermore, there existed an abnormal coronary flow reserve in the site distal to the bridged segment [8]. In case 3, the faster atrial fibrillation increased the demand and decreased the supply of myocardial blood flow, which intensified myocardial supply/demand mismatch and leaded to acute ischemia.

Sudden cardiac death is an uncommon type of coronary artery disease, which mainly caused by ventricular flutter or ventricular fibrillation. Corrado D et al. [9] reported that in their study, among 16 sudden cardiac deaths with aged under 35 years caused by non-atherosclerotic coronary diseases, six cases were founded to have MB in the LAD. In addition, MB can lead to sudden death among of the young athlete such as basketball and football players [10]. These studies indicated that MB may be a cause of sudden cardiac death among individuals without coronary atherosclerosis, which seemed superficially healthy. In case 4 of present study, during and after episodes of chest pain, the patient suffered several episodes of cardiac arrest, which may be associated with MB in the LAD.

The patients with MB can be treated according to the Schwarz classification [11]. No treatment was needed in patients without objective signs of ischemia. For symptomatic patients, beta-blockers and non-dihydropyridine calcium-channel blockers are important drugs in first-line therapy. Owing to intensifying systolic compression of the bridged segment, nitrates should be avoided unless there is significant concomitant vasospasm. Indeed, though increasing the milking effect on angiography, nitrates have been used effectively in some patients (such as case 2 in present study), which may be associated with their capability to reduce preload and to relieve vasospasm [12]. Intracoronary stenting, myotomy and coronary artery bypass graft surgery should be limited to patients with refractory symptoms despite intensified medical therapy.

## Conclusions

In conclusion, the present study showed that though MB is generally thought to be a benign coronary abnormality, it may lead to myocardial ischemia, myocardial infarction and even sudden death.

### Consent

Written informed consent were obtained from all patients for publication of the cases report and any accompanying images. A copy of the written consent is available for review by the Editor of this journal.

## Additional file

Additional file 1:
**ECGs with case 2 to 4 were showed.** (PDF 396 kb)

## Abbreviations

MB: Myocardial bridge
LAD: Left anterior descending coronary artery
ECG: Electrocardiogram
CAG: Coronary angiography
