# Supplementary material for: Myocardia ischemia associated with a myocardial bridge with no significant atherosclerotic stenosis
Source: BMC Cardiovasc Disord. 2015 Dec 8;15:165. doi: 10.1186/s12872-015-0158-2 (PMC4673761; doi:10.1186/s12872-015-0158-2)
Supplement: Additional file 1: — ECGs with case 2 to 4 were showed. (PDF 396 kb) [file 12872_2015_158_MOESM1_ESM.pdf]

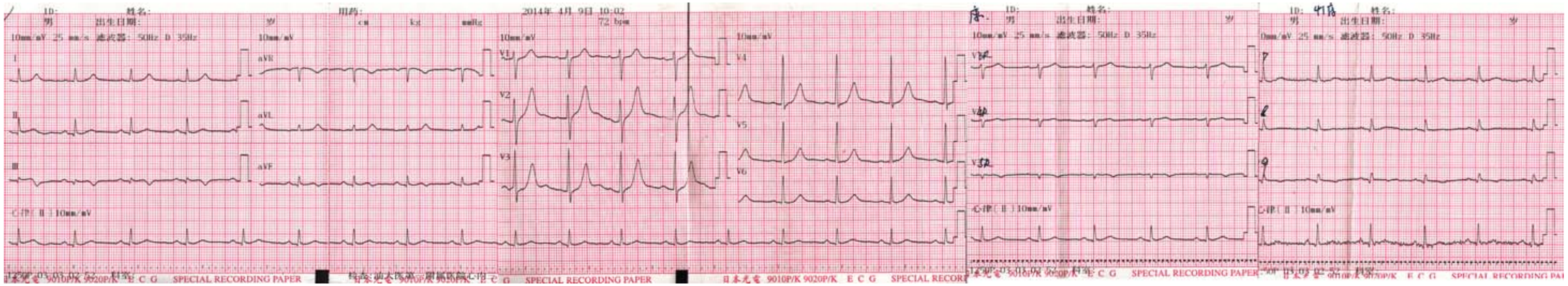

Figure S1. In Case 2, the patient was admitted because of a suddenly chest pain lasting about 80 minutes. ECG showed ST segment in III and aVF leads were slightly elevated. The levels of cardiac biomarkers were elevated. The levels of troponin I, creatine kinase-MB and myohemoglobin were 6.0 ng/ml, 43.5 ng/ml and 148 ng/ml respectively. The patient was diagnosed with acute coronary syndrome. CAG showed that a MB in the middle segment of the LAD and no stenosis of coronary artery.

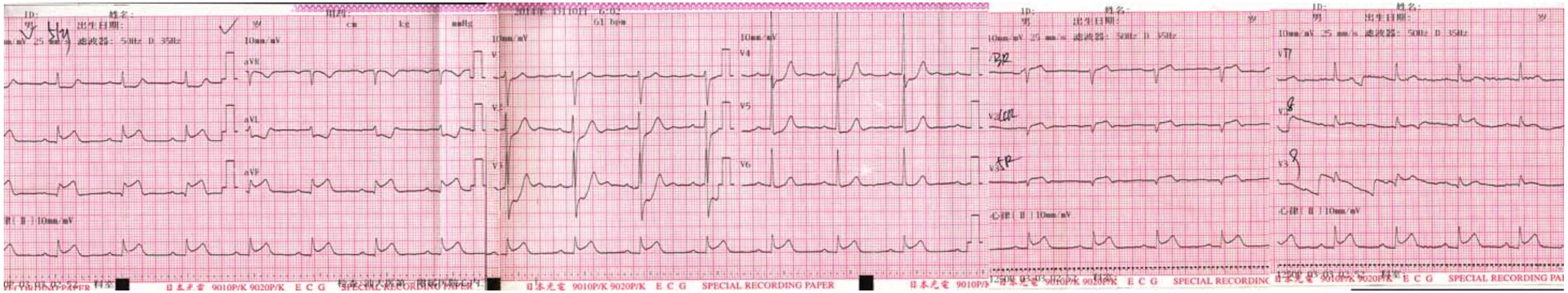

Figure S2. In Case 2, one day after CAG, the patient suffered episode of chest pain. ECG showed that ST segment elevation in II, III, aVF, V3R-V5R and ST segment depression in V2-V5 leads. After treatment with nitrates and diltiazem, the chest pain was relieved, which suggested that right coronary artery spasm may lead to episode of chest pain.

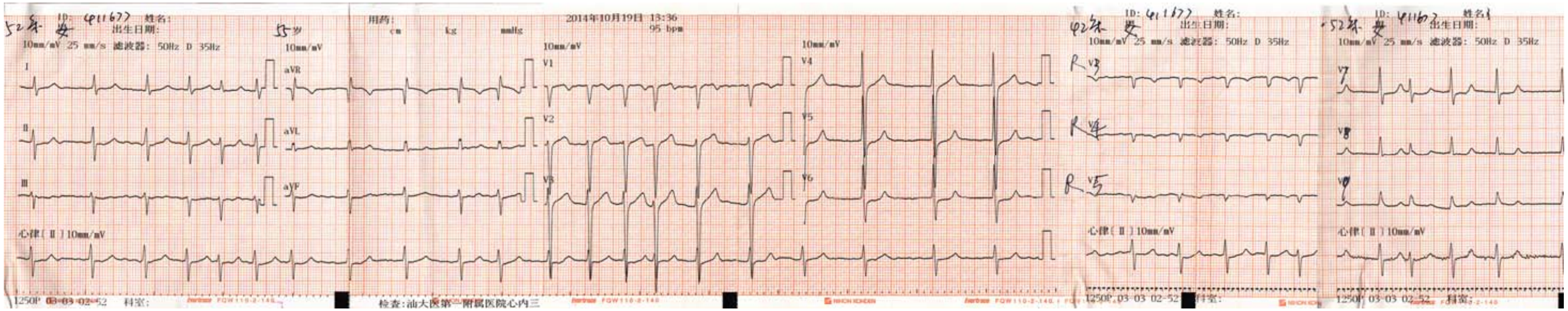

Figure S3. In Case 3, the patient was presented because of suddenly chest pain and shortness of breath for 3 hours. The ECG revealed that faster atrial fibrillation and ST segment depression in leads V6. The dynamic examination of cardiac biomarkers revealed that the levels of troponin I, and creatine kinase-MB increased. CAG showed that a MB in the middle segment of the LAD. No atherosclerotic lesions were found. Three days later, atrial fibrillation was converted to sinus rhythm.

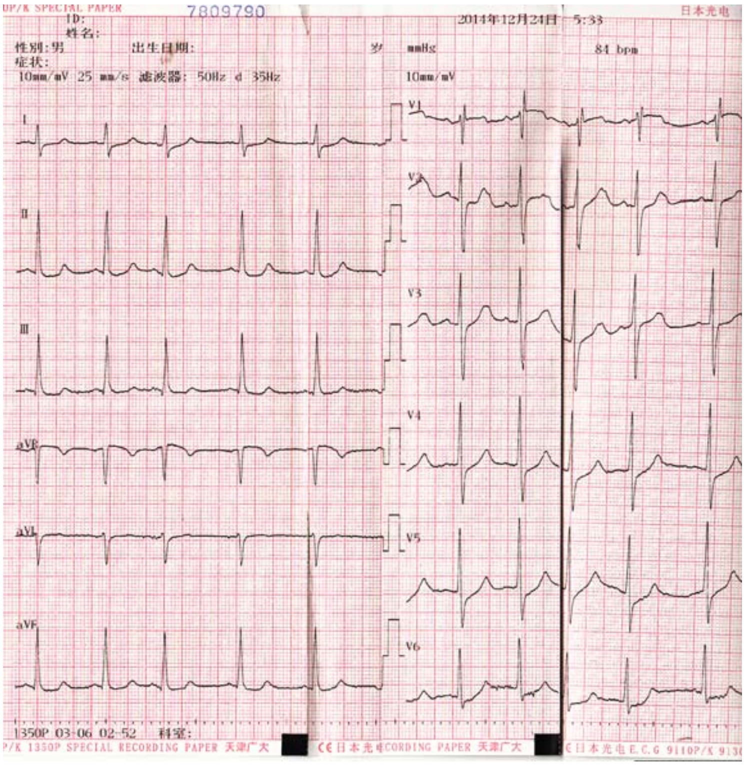

Figure S4. In case 4, the patient was send to our hospital for lasting chest pain for 6 hours and sudden cardiac arrest. On admission, ECG showed that ST segment depression in leads V4-V6. The levels of troponin I and creatine kinase-MB were normal. He suffered recurred cardiac arrest in hospital. CAG showed that a MB in the middle segment of the LAD.
